# Calcium and Vitamin D Supplement Prescribing Practices among Providers Caring for Children with Autism Spectrum Disorders: Are We Addressing Bone Health?

**DOI:** 10.1155/2016/6763205

**Published:** 2016-03-06

**Authors:** Shylaja Srinivasan, Julia O'Rourke, Sara Bersche Golas, Ann Neumeyer, Madhusmita Misra

**Affiliations:** ^1^Pediatric Endocrinology, Massachusetts General Hospital and Harvard Medical School, Boston, MA, USA; ^2^Lurie Center for Autism, Massachusetts General Hospital for Children, Boston, MA, USA; ^3^Pediatric Neurology, Massachusetts General Hospital and Harvard Medical School, Boston, MA, USA

## Abstract

Children with autism spectrum disorders (ASD) have several risk factors for low bone mineral density. The gluten-free, casein-free (GFCF) diet is a complementary therapy sometimes used in ASD that raises concerns for the adequacy of calcium and vitamin D intake. This study evaluated the prescribing practices of calcium and vitamin D supplements and the practice of checking 25-hydroxy vitamin D (25(OH)D) levels by providers in 100 children with ASD, 50 of whom were on the GFCF diet. Fifty-two percent and 46% of children on the GFCF diet were on some form of vitamin D and calcium supplements, respectively, compared to 18% and 14% of those not on this diet. Twenty-four percent of children in the GFCF group had a documented 25(OH)D level compared to none in the non-GFCF group. The data highlight a gap in calcium and vitamin D supplement prescribing practices among providers caring for children with ASD as well as a gap in the practice of checking 25(OH)D levels.

## 1. Introduction

Autism spectrum disorder (ASD) is a developmental disorder with various degrees of severity that can cause significant social, communication, and behavioral challenges. Data from the Center for Disease Control's Autism and Developmental Disabilities Monitoring (ADDM) Network show that the estimated number of children identified with ASD continues to rise in the United States with 1 in 68 children estimated to have an ASD in 2014 [1]. Peak bone mass, an important determinant of future bone health, is achieved by early adulthood, and the childhood and adolescent years are a critical period for bone accrual. Factors that affect bone accrual in childhood include genetics, nutritional status, calcium and vitamin D intake, weight bearing activity, hormonal alterations, and the use of certain medications [2]. Children with ASD have several risk factors that could potentially contribute to low bone mineral density (BMD), including decreased exercise, restricted diets, and use of medications that are deleterious to bone health. Nutritional status is a potentially modifiable factor and is very relevant to bone accrual in children. In the absence of curative options for the management of ASD, many parents turn towards alternative therapies and the gluten-free, casein-free (GFCF) diet is one such dietary therapy. A systematic review of studies published from 1970 to 2014 related to the GFCF diet in ASD patients showed that the evidence on this topic is currently limited and weak [3]. However, this diet continues to be used by children with ASD, largely based on anecdotal evidence for improvement.

In the US, most of one's dietary intake of calcium comes from dairy products while the dietary intake of vitamin D is derived from fortified foods such as milk and milk products with vitamin D [4]. In addition, children derive vitamin D through sun exposure. A diet limited in dairy products such as the GFCF diet raises concerns for the adequacy of calcium and vitamin D intake in the absence of supplementation. Additionally, outdoor physical activity and sun exposure may be limited in children with ASD compared to their typical peers limiting their endogenous vitamin D production, which is already limited in the northern latitudes. A low serum phosphorus level could be one of the initial signs of mild vitamin D deficiency that occurs even before serum calcium level falls [5].

Other risk factors for low BMD in children with ASD include reduced weight bearing activity and high rates of comorbid neurologic and psychiatric illnesses, including epilepsy and mood disorders. The latter may contribute to low BMD by increasing cortisol levels [6, 7]. Medications used to treat these conditions, including antiepileptics, antidepressants, and antipsychotic medications, also have influences on bone indices and vitamin D metabolism [7–12]. Finally, children with ASD are often on proton pump inhibitors, which may influence bone health by impairing insoluble calcium absorption.

We have previously examined the relationship between ASD and BMD in peripubertal boys [13]. In this study, boys with ASD had lower BMD at the hip, femoral neck, and lumbar spine compared to typically developing controls. BMD was even lower in boys with ASD on a GFCF diet. In a subsequent study that examined the prevalence of fractures in children and adults with and without ASD using a national database of emergency room visits, we found a higher odds ratio for hip fractures in children and young adults (3–22 years) as well as older adults (23–50 years) with ASD than those without ASD and a higher odds ratio for forearm and spine fractures in women aged 23–50 years with ASD [14].

With the knowledge that children with ASD have multiple risk factors for low BMD and with increasing evidence to show that individuals with ASD may have low BMD and increased risk of fractures, there is a compelling need to determine how physicians are addressing the issue of bone health in these children. Little is known about the prescribing practice of vitamin D and calcium supplements in this population or the practice of checking vitamin D levels in this group, particularly in children on the GFCF diet, who may be at higher risk. The primary objective of our study was to review the calcium and vitamin D supplement prescribing practices of physicians caring for children with ASD on a GFCF diet. Our secondary objectives were to review the practice of checking 25(OH)D and phosphorus levels in this group and to describe the use of medications in these children that may affect bone health.

## 2. Methods

### 2.1. Study Design and Subject Selection

This was a retrospective study of patients who presented to the Lurie Center for Autism at the Massachusetts General Hospital for routine clinic visits. The Lurie Center is a multidisciplinary clinical and research center dedicated to the care of individuals with autism spectrum disorders and other developmental disorders. The Lurie Center Data Repository (LCDR) obtains patient data via batched data transfer from the Partners Research Patient Data Registry (RPDR) into a secure, relational database, which is organized and queried using the Structured Query Language (SQL) Server Management Studio. Data available from the RPDR include patient demographics, encounter billing data, diagnosis billing data, outpatient clinical notes, and prescribed medications.

The patient population was identified by searching the database for individuals who had encounters at MGH with a billed diagnosis of ASD (“299” ICD-9 codes) when they were between 3 and 25 years of age and had been seen at the Lurie Center between 2004 and 2014, with a total of 2814 subjects identified. All subjects met Diagnostic and Statistical Manual of Mental Disorders (either IV or V) criteria for diagnosis of an ASD. Clinical notes in the longitudinal medical record for these patients were then reviewed for language referring to the patient being on a gluten and/or casein-free diet (e.g., “gluten-free,” “casein-free,” “dairy-free,” and “gluten and casein”). To do this, an SQL code was written that would extract portions of note text surrounding occurrences of the word “diet” which also contained the GFCF terms, excluding notes with terms such as “not on a gluten-free” or “not on a strict casein or gluten-free” diet. These clinical note extracts were then reviewed by study staff to determine which patients were on a dedicated GFCF diet. Using this strategy, a total of 837 subjects were identified to be on a GFCF diet. We excluded subjects who had a condition known to influence bone health such as celiac disease, inflammatory bowel disease, or renal disease. We then randomly selected 100 subjects for our study of whom 50 were on the GFCF diet and 50 were not. According to the Endocrine Society's Clinical Practice guidelines, we defined vitamin D deficiency as a 25(OH)D level below 20 ng/mL and vitamin D insufficiency as a 25(OH)D level of 21–29 ng/mL [15].

This protocol was approved by the Partners Health Care Institutional Review Board, and procedures used for data collection were in compliance with the Health Insurance Portability and Accountability Act (HIPAA). Due to the retrospective nature of the study, and in accordance with the guidelines of our Institutional Review Board, informed consent and assent were not obtained.

### 2.2. Data Collection

We collected the following demographic data: age, sex, race, and self-reported ethnicity. The clinical data that were collected included information regarding vitamin D and calcium supplementation, 25(OH)D and phosphorus levels, and use of antiepileptic, antipsychotic, and antidepressant medications and proton pump inhibitors. Information regarding Tanner stage of subjects was not available because the clinical information was collected at specialized neurodevelopmental visits. Similarly, subjects did not have parathyroid hormone (PTH) levels measured.

To determine vitamin D and calcium supplementation status, medication data was queried in the LCDR to find who in the study population had been prescribed vitamin D or calcium by their provider. Additionally, active and inactive medication lists were reviewed in the electronic medical record. Clinical notes were also queried using the same text extraction method described above for notes containing references to patients supplementing their diet with vitamin D or calcium, with or without a prescription from their provider. These note extracts were then reviewed by study staff to confirm that subjects were on supplements. For the purpose of our study, we counted the use of multivitamins towards supplementation of both vitamin D and calcium. When we surveyed some of the commonly used brands of children's multivitamins, we found that in general most brands tend to have around 600 IU of vitamin D and around 100 mg of elemental calcium. To determine vitamin D and phosphorus levels, the laboratory results section of the subject's electronic medical record was reviewed. Also, clinical note text extraction was performed for notes containing references to 25(OH)D and phosphorus level laboratory results with subsequent confirmation done by study staff. The same methods were used to determine the use of antiepileptic, antipsychotic, and antidepressant medications, or proton pump inhibitors in the study population.

### 2.3. Data Analysis

Data analysis was performed using JMP® statistical software version 11 (SAS Institute, Cary, NC). Analyses were performed using Student's *t*-test for continuous variables and Pearson's *χ* ^2^ test for categorical variables. A *p* value of less than 0.05 was considered to be significant.

## 3. Results

The clinical characteristics of children with ASD on a GFCF diet versus those not on a GFCF diet are summarized in Table 1. The groups did not differ for demographic characteristics, including age, sex, and self-reported ethnicity.

### 3.1. Vitamin D and Calcium Supplementation

**Figure 1:**
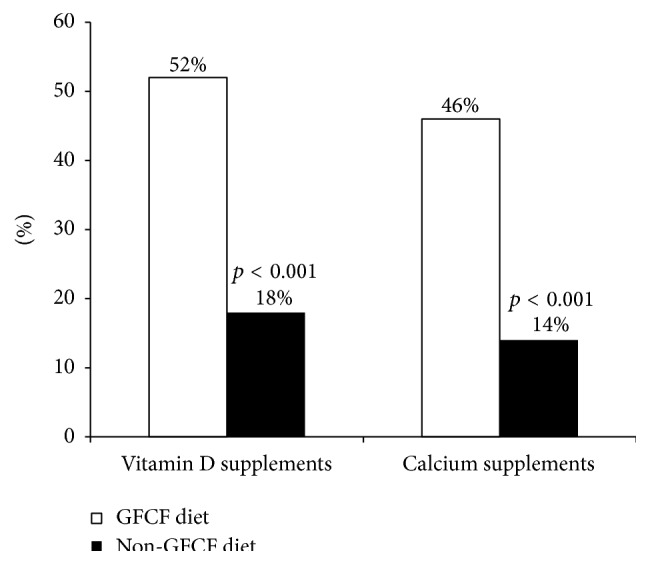
Percentage of children on the GFCF diet versus the non-GFCF diet taking vitamin D and calcium supplements.

Fifty-two percent of the children on the GFCF diet were taking vitamin D supplementation in comparison to 18% of those in the non-GFCF group. Forty-six percent of subjects who were on the GFCF diet were taking or prescribed calcium supplementation in comparison with 14% in the non-GFCF group (Figure 1). Compared to children on a non-GFCF diet, children on the GFCF diet were more likely to be taking vitamin D [OR 4.94, 95% CI (1.99–12.26), and *p* = 0.0006] and calcium supplements [OR 5.23, 95% CI (2.63–10.41), and *p* < 0.0001].

### 3.2. 25(OH) Vitamin D Levels and Phosphorus Levels

**Figure 2:**
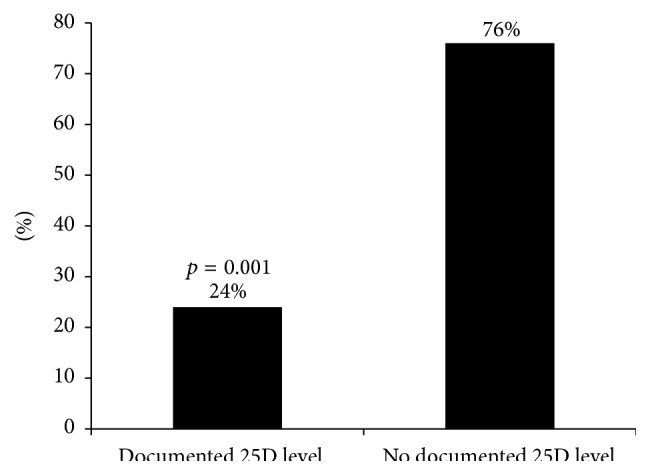
Percentage of subjects on the GFCF diet who had a documented 25(OH)D level.

**Figure 3:**
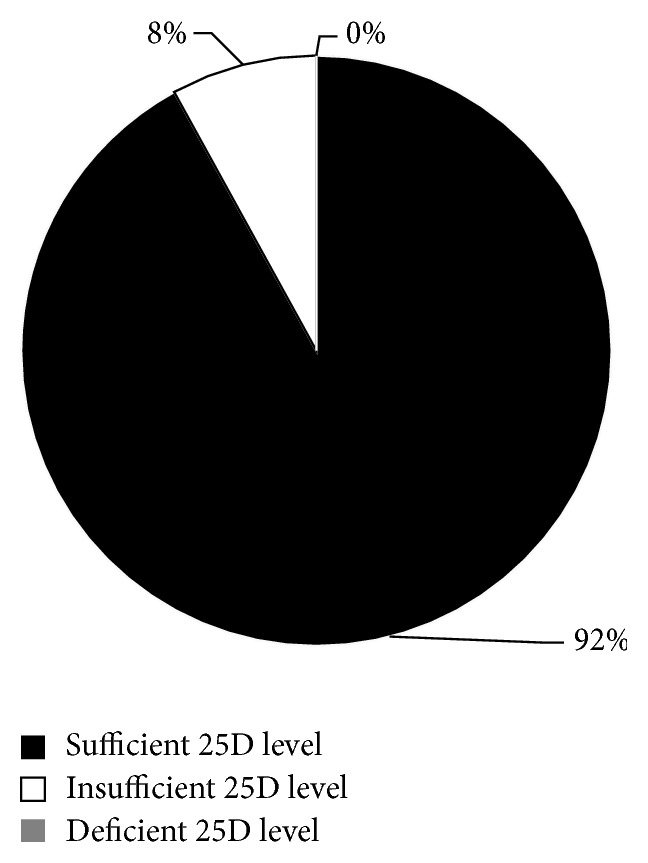
Proportion of subjects on the GFCF diet with documented 25(OH)D levels, with sufficient levels. In accordance with the Endocrine Society Practice guidelines [15], a sufficient level was considered to be ≥30 ng/mL, an insufficient level was considered to be between 21 and 29 ng/mL, and a deficient level was considered to be ≤20 ng/dL.

Twenty-four percent of children in the GFCF group had a documented 25(OH)D level compared to none in the non-GFCF group (Figure 2). The mean 25(OH)D level in children on the GFCF diet was 41.4 ng/mL (SD 11.4). Of subjects on the GFCF diet who had a 25(OH)D level checked, 92% had a level greater than 30 ng/mL, the lower limit for sufficiency as suggested by the Endocrine Society's Clinical Practice guidelines. Of the 24% of subjects on the GFCF diet with a documented 25(OH)D level, 83% were taking a vitamin D supplement (Figure 3). We further looked at the season in which the 25(OH)D levels were measured and it is worthy to note that the only two insufficient values were measured in the winter months. We were unable to make a statistical inference regarding seasonality because of our limited sample size. The mean vitamin D level was higher in the GFCF group that received supplementation (mean 42.8 ng/dL, SD 11.72) than in the group that did not (mean 32.5 ng/dL, SD 3.54). Sixteen percent of children on the GFCF diet had a documented phosphorus level in comparison to 2% on the non-GFCF diet. The mean phosphorus level when checked was 5.4 mg/dL (SD 1.0) in the GFCF group and 3.05 mg/dL (SD 0.5) in the non-GFCF group.

### 3.3. Medication Use

The use of antiepileptic, antipsychotic, and antidepressant medication was similar between the two groups. Some of the more commonly prescribed antiepileptic medications were topiramate, valproic acid, lamotrigine, and levetiracetam. The most commonly prescribed antipsychotic medications included risperidone, quetiapine, and aripiprazole. Fluoxetine, sertraline, and citalopram were the most commonly prescribed antidepressant medications. Children in the GFCF group were more likely to be on a proton pump inhibitor (*p* = 0.02).

## 4. Discussion

Our results suggest that, in both groups, a large proportion of subjects were not supplemented with calcium or vitamin D. Children who were on the GFCF diet were more likely to be supplemented with calcium and vitamin D than those not on the diet. However, even in the GFCF group, approximately 50% of the subjects did not receive supplementation with either nutrient. As stated earlier, for the purpose of this study, we counted the use of multivitamins towards both vitamin D and calcium supplementation as most children's multivitamins contain vitamin D and many contain calcium. Therefore, our results may even be an overestimate of the actual proportion of children with ASD receiving supplements.

Our results also indicate that only a small proportion of children on the GFCF diet and none on the non-GFCF diet had vitamin D levels checked, even when on multiple medications that could potentially affect vitamin D metabolism and bone health. Of note, a large proportion of the vitamin D levels that were checked were above 30 ng/mL, the recommended lower limit for sufficient levels according to the Clinical Practice guidelines of the Endocrine Society [15]. This suggests that those providers who were checking levels were also supplementing their patients with vitamin D, leading to sufficient levels in these children. Of note, serum phosphate levels may decrease slightly following an oral glucose load [16], a phenomenon that is thought to be mediated through insulin. However, we do not believe that this would significantly confound the serum phosphate results and clinically do not require patients to be fasting when measuring serum phosphorus levels.

Regarding medication usage, both groups of subjects were on multiple medications that could potentially affect bone health. Antiepileptic drugs such as phenytoin, primidone, carbamazepine, oxcarbazepine, ethosuximide, and phenobarbital induce the cytochrome p450 enzyme system. This induction leads to increased catabolism of vitamin D to inactive metabolites and a subsequent rise in parathyroid hormone (PTH) levels. The rise in PTH increases mobilization of calcium from bone and thereby modifies bone turnover [8]. In contrast, sodium valproate inhibits the cytochrome p450 system, and its use is also associated with abnormal biochemical indices of bone and mineral metabolism, bone loss, and higher fracture rates in adults and children [7, 9]. Few studies have examined the newer antiepileptic medications in relation to vitamin D and bone metabolism and fracture risk. The use of lamotrigine, increasingly common in children, is associated with lower BMD and higher markers of bone turnover compared to children not receiving antiepileptic medications [10]. Furthermore, atypical antipsychotics may impact bone by increasing prolactin secretion and causing hypogonadism [11]. Both tricyclic antidepressants and selective serotonin reuptake inhibitors are associated with an increased risk of fragility fractures in observational studies in adults [12]. Whether the effects of these medications on fractures are from direct effects of these drugs on bone or from indirect effects related to sedation and falls needs further investigation. A longitudinal study of 96 boys between the ages of 7 and 17 years showed that chronic SSRI treatment in children was associated with reduced but stable bone mass for age, while chronic risperidone treatment was associated with failure to accrue bone mass [17]. Drugs such as proton pump inhibitors (PPIs) that reduce gastric acid secretion may decrease calcium absorption because insoluble calcium such as in calcium carbonate requires an acidic environment for absorption. Whether PPI use is associated with increased fracture risk is yet to be definitively established.

To our knowledge, this is one of the first studies evaluating the prescribing practices of calcium and vitamin D by providers caring for children with ASD and the practice of checking vitamin D levels in these children. Stewart et al. in 2015 reported the results of a cross-sectional study in 288 children with ASD that examined dietary supplement use and micronutrient intake. A GFCF diet was consumed by 12% of participants. Their results showed that even after supplementation, about 50% of children with ASD had inadequate intake of calcium, and about 30% had inadequate intake of vitamin D. Similar to our results, their study showed that children on the GFCF diet were more likely to be on supplements [18].

One of the strengths of our study is that it was conducted in a specialized tertiary care outpatient center with access to a large number of patients with ASD on the GFCF diet. The use of the electronic medical record and SQL code to extract text for keywords ensured that a thorough chart review was completed. This study also has limitations that are inherent to a retrospective chart review. One such limitation is that we were unable to quantify the duration of exposure of subjects to the GFCF diet. However, because we looked at whether subjects were ever on vitamin D and calcium supplementation at our center, we likely also covered the period that they were on the GFCF diet. Another limitation is that parents may not have reported medication use, especially over the counter vitamins and supplements, and providers may not have accurately documented medications especially when considering drugs such as multivitamins or supplements.

## 5. Conclusions

This study highlights a significant gap in the practice of prescribing vitamin D and calcium supplements in children with ASD and the practice of checking vitamin D and phosphorus levels in this group. Children with ASD have several risk factors that may affect BMD that providers caring for these children need to be aware of. Given that the childhood years are critical for bone accrual, we believe that children with ASD should be screened for vitamin D status with 25(OH)D levels and serum phosphorus levels and assessed for vitamin D and calcium intake. Supplementation should be implemented based on clinical judgment after evaluation of risk factors. This is particularly necessary in children on restrictive diets such as the GFCF diet and those on medications that can have adverse effects on bone. Prospective studies are needed to examine the effects of the GFCF diet on bone health and incidence of fractures in individuals with ASD and the impact of supplementation on these endpoints.

## Figures and Tables

**Table 1:** Clinical characteristics of children on the GFCF diet versus those not on a GFCF diet.

	Gluten-free, casein-free (GFCF) diet	Non-GFCF diet	*p* value
Mean age in years (SD)	9.62 (4.66)	11.7 (4.66)	0.069
Sex (% male)	74	78	0.644
Self-reported ethnicity (%)	Non-Hispanic White: 66	Non-Hispanic White: 62	0.378
	Hispanic: 2	Hispanic: 10	
	African American: 4	African American: 4	
	Asian: 16	Asian: 4	
	Other: 12	Other: 20	
% on vitamin D supplements	52	18	*<0.001*
% on calcium supplements	46	14	*<0.001*
% with documented 25(OH)D level	24	0	*0.001*
Mean 25(OH)D level in ng/mL (SD)	41.4 (11.4)	N/A	N/A
% with documented phosphorus level	16	2	*0.013*
Mean phosphorus level in mg/dL (SD)	5.4 (1.0)	3.05 (0.5)	0.062
% on antiepileptic drugs	14	22	0.303
% on antipsychotics	14	28	0.087
% on antidepressants	18	32	0.108
% on proton pump inhibitors	30	8	*0.002*

## Abbreviations

ASD: Autism spectrum disorders
GFCF: Gluten-free, casein-free
BMD: Bone mineral density
AED: Antiepileptic medication
AP: Antipsychotic medication
AD: Antidepressant medication
PPI: Proton pump inhibitor
SQL: Structured Query Language
HIPAA: Health Insurance Portability and Accountability Act
LCDR: Lurie Center Data Repository
RPDR: Research Patient Data Registry
PTH: Parathyroid hormone.
